# The Impact of Financial Reward Contingencies on Cognitive Function Profiles in Adult ADHD

**DOI:** 10.1371/journal.pone.0067002

**Published:** 2013-06-20

**Authors:** Ivo Marx, Cornelia Höpcke, Christoph Berger, Roland Wandschneider, Sabine C. Herpertz

**Affiliations:** 1 Department of Psychiatry and Psychotherapy, University of Rostock, Rostock, Germany; 2 Department of General Psychiatry, University of Heidelberg, Heidelberg, Germany; University of Wuerzburg, Germany

## Abstract

**Objectives:**

Although it is well established that cognitive performance in children with attention-deficit/hyperactivity disorder (ADHD) is affected by reward and that key deficits associated with the disorder may thereby be attenuated or even compensated, this phenomenon in adults with ADHD has thus far not been addressed. Therefore, the aim of the present study was to examine the motivating effect of financial reward on task performance in adults with ADHD by focusing on the domains of executive functioning, attention, time perception, and delay aversion.

**Methods:**

We examined male and female adults aged 18–40 years with ADHD (n = 38) along with a matched control group (n = 40) using six well-established experimental paradigms.

**Results:**

Impaired performance in the ADHD group was observed for stop-signal omission errors, n-back accuracy, reaction time variability in the continuous performance task, and time reproduction accuracy, and reward normalized time reproduction accuracy. Furthermore, when rewarded, subjects with ADHD exhibited longer reaction times and fewer false positives in the continuous performance task, which suggests the use of strategies to prevent impulsivity errors.

**Conclusions:**

Taken together, our results support the existence of both cognitive and motivational mechanisms for the disorder, which is in line with current models of ADHD. Furthermore, our data suggest cognitive strategies of “stopping and thinking” as a possible underlying mechanism for task improvement that seems to be mediated by reward, which highlights the importance of the interaction between motivation and cognition in adult ADHD.

## Introduction

Attention-deficit/hyperactivity disorder is a multidimensional disorder that is characterized by developmentally inappropriate levels of activity, impulsivity, and inattentive behavior. ADHD affects 5–10% of school-aged children [1] and remains prominent in 3–5% of adults previously diagnosed with childhood ADHD [2], [3]. In recent years, causal models of ADHD shifted from explaining the phenotype of the disorder according to single core deficits [4], [5] towards multiple-pathway approaches, and current models also incorporate motivational/emotional pathways [6]–[9]. This development was stimulated by data that challenged the opinion that ADHD is exclusively caused by deficits in executive functions [4], [5]. Thus it has been shown that measures of executive functioning demonstrate only medium effect sizes and high intra-group variability [10], [11], and that executive dysfunctions are linked dimensionally to inattention rather than to hyperactivity/impulsivity [10], [12]–[13], which suggests that especially those with the inattentive subtype might be impaired. Additionally, the discriminatory value of single tasks measuring executive functions in differentiating between subjects with and without ADHD appears to be minimal [10], and the correlations between measures of executive functioning and ADHD symptoms are nominal [14]–[15], which suggests that these measures cannot completely account for ADHD symptom variability. Furthermore, it has been shown that executive dysfunctions are not specific to ADHD [16]–[17], and that non-executive domains are also impaired in subjects with ADHD [18]–[21]. As measures of cognitive functioning in the executive and non-executive domain are uncorrelated but are both associated with ADHD symptoms, which has been demonstrated particularly in the domain of motivation/emotion [22]–[24], these measures permit for the definition of distinct neuropsychological subgroups of patients (executive deficit only, non-executive deficit only, combined executive and non-executive deficits, and no impairment) [10].

One of the most influential models at present, the dual-pathway model [9], [25], specifies alterations in two pathways which cause the disorder's phenotype. The first pathway is a cognitive pathway and involves alterations in the executive circuit. These executive circuit alterations which comprise regions of the frontal cortex, especially the dorsolateral prefrontal cortex, lead to executive dysfunction and cause cognitive and behavioral dysregulation (e.g., poor planning, ineffective behavior monitoring, and impaired emotion regulation). Alterations in the second pathway, a motivational pathway, affect the reward circuit, which incorporates the orbitofrontal cortex, anterior cingulate, nucleus accumbens, and amygdala. These alterations lead to performance deficits in delay-rich and potentially rewarding contexts. In particular, a shortened delay reward gradient, i.e., an increased level of delayed reward discounting, prompts the patient to attempt to avoid delay. Depending on whether or not this opportunity is available, the patient is led to make immediate choices or enact immediate behaviors in an attempt to minimize the experience of delayed results. Again, these behaviors constitute the symptoms of the disorder; immediate choices confer impulsivity, acting on the environment to provide stimulation creates hyperactivity, and directing attention to task-irrelevant stimuli in the environment manifests as inattention. This dysfunctional motivational style with a higher-than-normal tendency toward immediacy is termed delay aversion and has been demonstrated in both children [26], [27] and adults [28] with ADHD. The dual-pathway model suggests that reward contingencies such as the proximity of the reward and reward valence [29] may influence task performance by modulating ADHD symptoms.

Another influential model, the cognitive-energetic model [8], suggests that state factors such as *activation* (tonic physiological readiness to respond) and *arousal* (phasic responses associated with actual stimulus processing) influence individual task performance. According to this model, effective cognitive functioning relies on an optimal energetic state wherein arousal and activation are optimally adjusted to actual task demands. When the adjustment is suboptimal, i.e., when under- or overactivation occurs, performance deficits are predicted to occur. Adjustments to the actual task demands are provided by the *effort* pool, which increases or decreases activation and arousal. In subjects with ADHD, deficient functioning of the effort pool is assumed to cause energetic maladjustment and, as a consequence, impaired task performance. Support for the cognitive-energetic model has been largely derived from the literature on event rate effects, i.e., the speed with which stimuli are presented (for reviews, see [30], [31]). Based on these findings, it has been proposed that best performance will be obtained at medium event rates. When the event rate is low, patients with ADHD exhibit slow and inaccurate responses, and when the event rate is high, patients exhibit fast and inaccurate responses. However, only a few studies have tested this inverted U-shaped performance pattern directly, and most of these studies [31], though not all [32], seem to support model predictions, especially for low event rates. Similarly, analyses of reaction time variability in the frequency domain revealed a greater amplitude for intra-individual reaction time fluctuations in low frequency ranges (0.07 and 0.2 Hz) across different tasks in children with ADHD compared with controls [33]. It has been argued that these behavioral fluctuations may be related to fluctuations of (neuro)physiological processes [34]–[36].

Thus, the cognitive-energetic model describes suboptimal performance in ADHD as a response modulation deficit that is triggered by contextual factors such as event rate. Because effort is thought to be sensitive to motivation [30], reward constitutes a further contextual factor that has the potential to improve task performance by optimizing energetic states. In this sense, experiments manipulating stimulus event rates and reward conditions suggest that response time variability may be a key target variable that is susceptible to reward. When reaction time performance under low event rates was contrasted with that under fast, incentivized conditions, a higher event rate in combination with reward normalized the initially slower and more variable reaction times in children with ADHD. This effect was caused by a greater improvement in reaction time and a greater decrease in reaction time variability in the ADHD group compared with the control group [37], [38].

The dynamic developmental theory [7] explains altered reinforcement mechanisms in ADHD. According to this model, ADHD symptoms are caused by the hypofunctioning of the dopaminergic system and the subsequent altered reinforcement of novel behaviors and slower extinction of inadequate behaviors. As a result, subjects with ADHD fail to develop adaptive behavior in terms of long integrated behavioral chains and rather demonstrate impulsive, disorganized and highly variable behaviors that comprise the ADHD phenotype. Consistent with the dual pathway model [25], altered reinforcement mechanisms are described in terms of a steeper and shorter delay-of-reinforcement gradient. As a result, the time available for associating behavior with its consequences is shorter in ADHD than in normal children, and reinforcers (consequences) outside this narrow time window will not be effective. With regard to the delivery of the reward, the model suggests that reinforcement has to be delivered immediately and frequently to be effective.

With regard to the potential effects that reward may have on performance according to the above-mentioned models, the dual pathway model [25] and the dynamic developmental theory [7] predict that subjects with ADHD will perform best under immediate and frequent reinforcement [39]–[41], with frequency being more relevant than magnitude [42]. The cognitive-energetic model, in contrast, assumes an inverted U-shaped function rather than a linear function. Thus, the cognitive-energetic model predicts the best performance when contextual factors, such as reward frequency, reward magnitude, or task-associated delay, are of medium strength, thereby promoting an optimal energetic state [8], [42]. Despite these differences in detail, however, all three models exhibit motivational pathways for ADHD, assuming that subjects with ADHD will benefit from rewarding conditions.

Thus, if motivational factors significantly affect task performance in subjects with ADHD, subjects with ADHD should exhibit cognitive deficits in the absence of motivational stimulation compared with controls, and motivational stimulation should improve the task performance of ADHD patients. Therefore, the lack of improvement would suggest a “pure” cognitive deficit, partial improvement would be expected for a combined motivational and cognitive deficit, and compensation of performance deficits would demonstrate a “pure” motivational deficit.

### Cognitive Dysfunction Profile in Adult ADHD

#### Inhibition

The ability to inhibit prepotent motor responses has been argued to be one of the core deficits in ADHD [43]. One of the most widely used tasks to examine inhibitory control in patients with ADHD is the stop-signal task [44], which requires the patient to respond as quickly as possible to a target stimulus but to withhold the response when a stop signal is presented in a varying time interval shortly after the presentation of the target stimulus. Measurements derived from this task include the reaction time to the target stimulus in trials where no stopping is required (RT), the reaction time variability (SDRT) in those trials, the stop-signal reaction time (SSRT) (i.e., the time needed to stop the initiated motor response), and the SSRT relative to RT as an indicator of an abnormally prolonged stopping process. Measures of accuracy are also examined, including the number or proportion of successful inhibitions and omission errors. These measures are explained in more detail in Logan [44] and Verbruggen & Logan [45]. Meta-analyses indicate a slower RT and higher SDRT as well as prolonged SSRT in children with ADHD [16], [46], with no abnormalities observed for SSRT relative to RT. In contrast, adults with ADHD were not observed to have longer RT, but they exhibited higher SDRT and prolonged SSRT as well as abnormally prolonged SSRT relative to RT [46]. Based on their meta-analytical findings [46], the authors therefore assume that in children, impaired stop-signal task performance may primarily be based on attention deficits (slow and variable responding, slow stopping), whereas the inhibitory deficit itself may be more prevalent in adults with ADHD (no effect on RT but disproportionally elongated SSRT relative to RT). Another meta-analysis [19] and further studies [47], [48] observed SSRT to be prolonged in adult ADHD as well. By contrast, the evidence for RT differences appears to be inconclusive, as it supports slower [47], [48], equal [46], and faster RT [19]. In addition to differences in the speed components of the stop-signal task, analyses of accuracy measurements suggest that children with ADHD are impaired in the probability of inhibition [16], whereas adults do not seem to exhibit impaired performance accuracy in terms of the number or proportion of successful inhibitions [47], [49]–[52] but seem to commit more omission errors than controls [47].

#### Working memory

The term working memory refers to a temporally and quantitatively limited storage mechanism in which information is actively processed (i.e., stored, monitored and manipulated) to provide complex goal-directed behavior. In adults with ADHD, meta-analyses have revealed working memory deficits [18], [20] that seem to be more distinct in the spatial than in the verbal domain [11]. A spatial working memory task that captures increasing attention in adult ADHD research is the n-back task. Subjects are shown, in quick succession, sequences of visual stimuli and asked to decide whether the displayed stimulus matches the one that was presented *n* positions before, with task difficulty varying as a function of *n*. Measurements that can be derived from this task include RT, SDRT, and performance accuracy, which can be a single measurement (e.g., true positives or “hits”; false positives or “false alarms”) or a composite measurement (e.g., discriminability score d′ that takes both hits and false alarms into account). Using the n-back task, two studies found performance accuracy to be unimpaired in adults with ADHD [28], [53], and one study found performance accuracy to be impaired in adults with ADHD [48]. Although the RT for correctly identified targets did not differ between experimental groups in the study by Marx et al. [28], the overall RT was prolonged in the study by Bayerl et al. [54]. One of the studies cited did not report measurements of performance accuracy [54], and two studies did not analyze the RT [48], [53]. To the best of our knowledge, no studies to date have analyzed SDRT.

#### Sustained attention

The term sustained attention denotes the maintenance of attention allocation over a longer period of time. One of the most typically applied tasks in the domain of sustained attention is the continuous performance task (CPT), which requires the patient to detect a target sequence that is embedded in randomly presented visual stimuli. Variables of interest include measurements of performance accuracy, omission errors, RT, and SDRT. Four meta-analyses addressed CPT performance in adults with ADHD and consistently found that subjects with ADHD performed less accurately and exhibited a higher number of omission errors and higher SDRT, whereas no RT differences were observed between the groups [11], [18], [19], [21]. Omission errors occurred more often when the frequency of target stimuli was low [19].

#### Time Discrimination

Time discrimination tasks evaluate the ability of a patient to discriminate between stimuli that differ in their presentation time for only several milliseconds. Dependent variables of interest include the performance quality (i.e., the number of correct decisions in choice paradigms; the sensitivity threshold in adaptive paradigms, i.e., the point at which a subject fails to discriminate the presentation time of both stimuli adequately and reports them as equal), and the time needed to make a decision. Discriminatory deficits have been consistently observed in children and adolescents with ADHD [28], [55]–[57], as well as in adults with ADHD [28], [57]. Valko et al. [57] observed a differential pattern of task processing in children and adults with ADHD. Although children did not differ from controls with regard to decision time but made fewer correct decisions, adults were slower than controls but performed equally well. This behavior could reflect strategy use; adults took more time than children to make a decision, thereby resulting in a better performance quality.

#### Time Reproduction

In this type of task, stimuli are presented for a defined time interval (usually in the range of 2 to 60 seconds), and participants have to infer the duration of stimulus presentation and indicate this time interval by pressing a button. Absolute discrepancy scores (i.e., the absolute value of the deviation between the specified and the produced time interval), which reflect the overall magnitude of error regardless of its direction, and accuracy coefficient scores (i.e., the produced time interval divided by the specified time interval), which reflect the under- and over-reproductions, are used as dependent variables. Compared with controls, children and adolescents with ADHD seem to overestimate short time intervals and underestimate long time intervals [28], [57]–[59]. They also appear to reproduce larger discrepancies with increasing interval length [60], [61], which results in higher absolute discrepancy scores. Thus far, only two studies have examined time reproduction in adults with ADHD. Marx et al. [28] determined that children, adolescents and adults with ADHD exhibit higher absolute discrepancy scores compared with controls and produce larger discrepancies with increasing interval lengths. Notably, the latter finding was attributed to the underestimation of long time intervals by ADHD adolescents and adults but not children with ADHD. Similarly, Valko et al. [57], using a time reproduction task with short time sequences (up to 8 seconds), observed decreased accuracy coefficient scores and higher reproduction variability in children and adults with ADHD.

#### Delay Aversion

As outlined above, the term delay aversion denotes a motivational tendency to avoid delays over time, with a preference for the immediate delivery of consequences to reduce waiting. The Choice Delay Task [22] and its variants were used in most of the studies conducted thus far. Using the Choice Delay Task, several studies have demonstrated that children and adolescents with ADHD choose small, immediate rewards more often than large, delayed rewards [22], [26]–[28], [62]–[70], but only one study thus far has demonstrated impairment in adult ADHD [28]. Recently, Müller et al. [71] presented a game-like paradigm that we adopted for our study and that, to the best of our knowledge, has not yet been established in adult ADHD research.

To summarize, the inhibitory deficit in adults with ADHD appears to be most prominent in terms of an abnormally prolonged stopping process, and sustained attention appears to be most affected in terms of impaired performance accuracy. Furthermore, both domains are characterized by increased reaction time variability, whereas reaction time itself appears largely unaffected. For the remaining domains in focus, the body of literature is less substantial. Research on working memory performance using the n-back task is deficient in the fact that most studies report measures of either performance accuracy or speed, complicating the interpretation of heterogeneous findings; however, both speed and accuracy measures were found to be impaired. In time discrimination tasks, adults with ADHD appear to perform less accurate but performance strategies of slowing might help the subjects to overcome their deficits, and in time reproduction tasks, they seem to underreproduce especially long time intervals which results in decreased reproduction accuracy. Furthermore, adults with ADHD seem to prefer small immediate over large delayed rewards.

### Experimental Findings on Rewarded Task Performance in ADHD

Although there are no pre-data on rewarded task performance in adults with ADHD, a performance-enhancing effect of reward contingencies has been found in studies in children and adolescents with ADHD (see Table 1). Summarizing the literature available, the implementation of reward seems to improve the performance in children with ADHD and in controls largely to the same extent. This has been demonstrated most consistently in terms of decreased SSRTs in the stop signal task [72]–[75] and was also found for CPT perceptual sensitivity which improved in the reward conditions [77], as well as for stop-signal RT [73], stop-signal SDRT [74] and time reproduction error [58] which decreased in the reward conditions. Group x condition interactions were observed for SSRT and RT in the stop-signal task [72], [73] and for absolute discrepancy scores in time reproduction [58], which indicates that children with ADHD benefited from rewards to a greater degree than controls and even equaled the performance of controls with regard to SSRT [72].

**Table 1:** Rewarded Task Performance in Children and Adolescents with ADHD*.

Paradigm	Number of Studies	Reward Type	Main Effect of Group	Main Effect of Condition	Group × Condition Interaction Effect
**SST**	n = 5 ^[72]–[76]^	reward ^[72]–[74]^	***SSRT*** analyzed in 5/5 studies ^[72]–[76]^		
		response cost ^[75], [76]^	ADHD>CON [^72]^, [74]–[76]	no reward > reward ^[72]–[75]^	subjects with ADHD improved more strongly than controls and normalized in the reward condition ^[72]^
			***RT*** analyzed in 3/5 studies ^[73], [74], [76]^		
			ADHD<CON [^73^]	no reward < reward ^[73]^	subjects with ADHD slowed down more strongly than controls in the reward condition ^[73]^
			***SDRT*** analyzed in 3/5 studies ^[73], [74], [76]^		
			ADHD>CON [^73]^, [74], [76]	no reward > reward ^[74]^	–
**CPT**	n = 2 ^[77]–[78]^	reward ^[77]–[78]^	***d′*** analyzed in 1/2 studies ^[77]^		
			ADHD<CON [^77^]	no reward < reward ^[77]^	–
			***OE*** analyzed in 1/2 studies ^[78]^		
			ADHD>CON [^78^]	–	–
**Time Reproduction**	n = 1 ^[58]^	reward	***ERR***		
			ADHD>CON	–	subjects with ADHD improved more strongly than controls in the reward condition

*Studies are cited which (1) included a control group, (2) contrasted reward and non-reward conditions, (3) used real rewards in terms of money or small gifts, and (4) discontinued medication in the ADHD group.

SST, stop-signal task; CPT, continuous performance task. SSRT, stop-signal reaction time; RT, reaction time in the “go”-trials; SDRT, standard deviation of reaction time in the “go”-trials; d′, performance accuracy; OE, omission errors; ERR, absolute value of the deviation between specified and produced time interval in milliseconds.

Delay aversion has extensively been examined within reward paradigms, but no study thus far has compared rewarded and non-rewarded conditions directly. Studies in which children received physical rewards (e.g., small gifts, sweets, and money) [22], [26], [27], [62]–[64], [66]–[69] and studies in which only reward feedback was given or the type of reward was not specified [28], [65], [68], [70] predominantly report a preference for small, immediate rewards over large, delayed rewards in children with ADHD, and only four studies observed no group differences between children with ADHD and controls [29], [79]–[81]. No published study to date has examined the influence of rewards on working memory and time discrimination in ADHD. Because these domains reflect key domains that are affected in individuals with ADHD and contain target variables known to be susceptible to reward (RT, SDRT, and performance accuracy), they were included in the present study.

Taken together, several studies on the influence of reward contingencies on cognitive task performance in children and adolescents with ADHD are available, and these studies suggest that parameters of response speed and homogeneity [37], [38], [72]–[76] and parameters of performance quality [58], [77], [78] may be influenced by incentives. However, the whole range of key cognitive domains affected in ADHD has not yet been examined for susceptibility to rewards. Furthermore, most of the studies conducted used only one or two tasks rather than a range of tasks and, in doing so, varied in the complexity of the dependent variables that were examined, which makes it difficult to analyze complex performance patterns that incorporate measurements of speed, homogeneity, and accuracy within a sample of affected subjects across different domains. Furthermore, no published study has examined the influence of reward contingencies on cognitive task performance in adults with ADHD. Given the neuroanatomical [82] and neurofunctional alterations in the reward system in children [78], [83] and adults [84], [85] with ADHD that point to an increased sensitivity to reward and decreased activity of regulatory mechanisms during rewarded task performance and in response to the receipt of anticipated reward, we expect an increased susceptibility to rewards to be a common characteristic independent of age.

The aim of the current study was (1) to identify core cognitive deficits in adult ADHD that have been repeatedly reported in children and adolescents with ADHD and (2) to use well-established paradigms for these domains to examine the influence of financial reward contingencies as potentially motivating factors for relevant performance parameters in adults with ADHD. We followed a task-oriented approach, which allowed the direct comparison of rewarded task performance patterns in children and adults with ADHD. Simultaneously, implementing a substantial number of tasks across several key domains of cognitive functioning allowed us to follow a parameter-oriented approach; we were able to determine which task parameters are the most susceptible to motivational incentives across domains. With regard to the dependent variables, we therefore considered a holistic approach and incorporated parameters of accuracy, speed, and reaction time variability. We are convinced that this approach best reflects the possible patterns of task performance (e.g., performing a task in a very fast and error-prone manner), as well as the possible changes in task performance strategies that may be induced by incentives (e.g., working more slowly and thus committing fewer errors). Based on the literature described above, the cognitive domains of interest for this study incorporate executive functioning (inhibition and working memory), attention (sustained attention), time perception (time discrimination and time reproduction), and delay aversion.

We hypothesized that subjects with ADHD in the non-rewarded condition would exhibit deficits in all cognitive domains examined and that they would demonstrate improved stop-signal task performance, CPT performance, and time reproduction performance in anticipation of a reward. Because group × condition interaction effects have been demonstrated for stop-signal task parameters and time reproduction parameters as outlined above, thus demonstrating that children with ADHD benefit more from rewards than controls, we anticipated that the performance improvements would be most pronounced for the stop-signal task and the time reproduction task. To the best of our knowledge, no study thus far has examined the influence of reward on working memory, time discrimination and delay aversion, but we anticipate the performance-improving effect of rewards to be present in these tasks as well.

## Methods

### Participants

We examined males and females with ADHD who were aged 18–40 years, together with gender-, age-, and IQ-matched controls. Patients were recruited from the ADHD outpatient service of the Department of Psychiatry and Psychotherapy, University of Rostock. Patients were screened for exclusion criteria by telephone before being invited to participate in the study. The detailed diagnostic procedure included German versions of the Barkley Interview [86], the Wender-Reimherr Interview [87], and German versions of the following questionnaires: a short version of the Wender Utah Rating Scale (WURS-k) [88], which was used to quantify ADHD symptoms in childhood; the Conners' Adult ADHD Rating Scales (CAARS-S:L) [89] and a short self-rating questionnaire based on the DSM-IV criteria (ADHS-SB) [90], which were used to assess current ADHD symptoms; and the Barratt Impulsiveness Scale (BIS-10) [91]. Adults also underwent extensive psychiatric examination using the SCID-I and II and a short version of the German Wechsler Adult Intelligence Scale - Revised (HAWIE-R) [92]. ADHD diagnoses, which were assigned by a senior clinical psychiatrist, included the following criteria: a WURS-k sum score ≥30 points [93], an age- and gender-adjusted total ADHD symptom subscale score ≥1.5 SD above the mean in the CAARS-S:L, substantial impairment in more than one setting, and clinically relevant psychological strain. University hospital employees and students who were personally contacted or recruited via announcements in the university hospital served as controls. Controls underwent the same diagnostic procedure as subjects with ADHD. Exclusion criteria for all participants included IQ≤85, neurological or endocrine disorders known to affect brain function, a previous head injury, a current mental disorder (schizophrenia, depression, anxiety disorder, and substance abuse or dependency) and borderline personality disorder (BPD).

Fifty-one subjects with a clinical diagnosis of ADHD and 43 controls were screened for participation in the study. According to the exclusion criteria, eight subjects with borderline personality disorder, one subject with epilepsy and tic disorder, and four subjects with subclinical symptom severity were excluded from the ADHD group. From the control group, one subject with epilepsy was excluded, and two additional subjects were excluded as a result of the age- and IQ-matching procedure. The final sample consisted of 38 subjects with ADHD (22 males, 16 females) and 40 controls (19 males, 21 females). With respect to ADHD subtypes, 30 subjects met the criteria for the combined subtype, seven subjects were primarily inattentive, and one subject was primarily hyperactive-impulsive. Eighteen ADHD subjects were drug-naïve; the others had previously taken methylphenidate but were free of any medication for a minimum of 72 h prior to testing. Within the ADHD group, the following comorbidities were present: two subjects with recurrent depressive disorders, currently remitted; one subject with an adjustment disorder; two subjects with a binge-eating disorder; and nine subjects with personality disorders (except for BPD). Within the control group, no psychiatric or personality disorders were observed. Subjects with ADHD were older but did not differ from the controls in terms of IQ. It should be noted that the relatively high IQ values could have resulted from the use of the short version of the WAIS, which utilizes relatively old German norms. We did not examine a group of only well-educated subjects; rather, educational and occupational levels were mixed in both experimental groups. Basic demographic and clinical sample characteristics are provided in Table 2.

**Table 2:** Sample Characteristics.

	ADHD R−	ADHD R+	CON R−	CON R+	Diagnostic Group	Reward	Diagnostic Group X Reward
	M (SD)	M (SD)	M (SD)	M (SD)			
	**(n = 18)**	**(n = 20)**	**(n = 20)**	**(n = 20)**		F(1, 74)	
**Age**	27.72 (6.21)	29.31 (6.58)	24.75 (3.63)	25.13 (5.43)	8.06**	0.61	0.23
**IQ**	123.33 (16.82)	128.10 (17.29)	127.65 (12.33)	125.45 (15.28)	0.06	0.13	0.98
**CAARS-S:L**	**(n = 16)**	**(n = 19)**	**(n = 20)**	**(n = 20)**		F(1, 71)	
** IN**	93.72 (11.50)	92.95 (21.72)	36.44 (32.77)	43.23 (28.90)	80.81***	0.26	0.40
** HI**	83.16 (17.04)	75.38 (33.96)	26.42 (28.42)	24.85 (26.41)	70.71***	0.54	0.24
** TOT**	93.93 (8.51)	89.99 (24.01)	26.27 (28.13)	28.57 (28.58)	130.89***	0.02	0.31
**ADHS-SB**	**(n = 20)**	**(n = 20)**	**(n = 18)**	**(n = 20)**		F(1, 74)	
** IN**	5.33 (2.20)	5.90 (2.86)	0.70 (2.03)	0.40 (0.75)	113.12***	0.08	0.83
** HI**	4.17 (2.55)	5.25 (3.63)	0.80 (1.91)	0.85 (1.18)	47.62***	1.01	0.84

ADHD, ADHD group; CON, control group; R−, no financial reward; R+, financial reward. Diagnosis, main effect of diagnostic group; Reward, main effect of reward condition; Diagnosis × Reward, interaction effect of diagnostic group and reward condition. n, number of subjects; M, median; SD, standard deviation; F, F-values. CAARS-S:L subscales (PR): IN, DSM-IV Inattentive Symptoms; HI, DSM-IV Hyperactive-Impulsive Symptoms; TOT, DSM-IV Total ADHD Symptoms. ADHS-SB subscales: IN, number of fulfilled criteria for inattention (DSM-IV); HI, number of fulfilled criteria for hyperactivity/impulsivity (DSM-IV). *p<0.05; **p<0.01; ***p<0.001.

All subjects were randomly assigned to the reward conditions (no performance-associated reward; performance-associated reward). Gender was equally distributed in the resulting four experimental groups, *χ* ^2^ = 1.09, *p* = .78, *ns* (ADHD, non-reward condition: 11 males, 7 females; ADHD, reward condition: 11 males, 9 females; controls, non-reward condition: 9 males, 11 females; controls, reward-condition: 10 males, 10 females). The ADHD subtype distribution and medication status was as follows: ADHD, non-reward condition: 13 combined, 4 inattentive, 1 hyperactive-impulsive, and 11 drug-naïve; ADHD, reward condition: 17 combined, 3 inattentive, and 7 drug-naïve.

### Tasks and Measures

#### Stop-Signal Task

The stop-signal task was designed based on the task of Carter et al. [94], although we used arrows as stimuli. The task consisted of 456 randomly presented arrows pointing to the left or to the right. In 160 of these trials, we presented stop signals subsequent to arrow presentation that occurred at four different proportions of “go”-RT (20%: 16 arrows; 40%: 64 arrows; 60%: 64 arrows; 80%: 16 arrows). As described in Carter et al. [94], the stop-signal task was preceded by a choice reaction time task consisting of 60 arrows (30 pointing to the left and 30 pointing to the right) that was used to compute the individual reaction times as an initial value for the first stop-signal task block. The following variables served as dependent measures: SSRT, median reaction time (MDRT), SDRT in the go-trials, number of correct inhibitions, and omission errors. For a detailed description of the task and measures, see Carter et al. [94].

#### N-back Task

Altogether, 12 n-back blocks (six 1-back, six 2-back) were randomly presented. Each block contained 14 letters (4 targets, 10 distractors) and was preceded by the numbers “1” or “2”, which indicated the respective n-back condition. The trial length was 3,000 ms. After 1,250 ms had elapsed, the n-back letter was presented for 500 ms. Consequently, subjects had a time window of 1,750 ms to respond. All subjects performed two 1-back and two 2-back practice trials prior to the task. The reaction time for correctly identified targets (RT) and performance accuracy (d′) served as dependent variables. Performance accuracy was analyzed based on signal detection theory, in which individual discrimination indices were calculated as follows: [d′  =  z (hits/number of targets) − z (false alarms/number of distractors)].

#### Continuous Performance Task (CPT)

In this task, 525 letters are randomly presented on the screen, and the participants have to press the left mouse button as fast as possible if the letter “X” appears subsequent to the letter “A”. The task comprised 52 target-sequences (“A–X”) and 20 “A” without “X” to measure omission errors. Each letter was presented for 250 ms, and the inter-stimulus interval was 1,500 ms. The task was preceded by a practice trial that contained 30 letters and three targets. False positives (FP), omission errors, MDRT for correctly identified targets and SDRT served as dependent variables.

#### Time Discrimination Task

Participants were shown a red circle and a green circle in quick succession that barely differed in presentation duration. The participants were then required to decide which of the circles was presented for a longer duration. One of the circles was always presented for 1,000 ms; the other circle was initially presented for 1,300 ms. Correct answers were followed by a 15-ms reduction in the presentation duration, whereas incorrect answers were followed by an increase of 15 ms. For task details, see Smith et al. [56]. The sensitivity threshold served as a dependent variable.

#### Time Reproduction Task

The ability to estimate a several-second interval of time was assessed using a time reproduction paradigm [95]. Subjects observed yellow smiley faces on the screen for a certain time interval (2, 6, 12, 24, 36, 48 or 60 s). They then had to infer the duration for which the smiley face was presented on the screen and were asked to press the left mouse button to indicate this time interval. During the button press phase, a green smiley face was displayed on the screen. In the inference and production phases, subjects were explicitly instructed to count the seconds non-verbally. The time intervals were presented twice, in two successive blocks, and their order was randomized within the blocks. The presentation of the smiley face was signaled by a 3-s countdown. Absolute discrepancy scores and accuracy coefficient scores were computed as dependent variables. With regard to accuracy coefficient scores, a score higher than 1.00 indicated over-reproduction, and a score lower than 1.00 indicated under-reproduction.

#### Delay Aversion Task

The applied delay aversion task was a modified version of the continuous delay aversion test introduced by Müller et al. [71]. In each of 22 subsequent trials, subjects were presented with a donkey whose mouth deposited gold into a basket. The amount of gold being delivered decreased over the course of the trial, and after 60 s, the distribution of earnings stopped, but waiting time was still registered. The trial duration was non-limited, and subjects decided at the push of a button when to complete the ongoing trial and obtain the next donkey. The subjects were instructed to collect as much gold as desired. Each trial was followed by a feedback trial that informed the subject of the number of trials remaining. For a more detailed task description, see Müller et al. [71]. The mean trial duration in seconds in the first and the second half of the experiment was used as the dependent variable. Subjects with ADHD have been demonstrated to avoid delay more strongly than controls if the experimental trial duration is not fixed, and ADHD subjects are not more delay-averse under conditions where the trial duration is fixed. However, it is unknown how an unavoidable preceding delay affects subsequent delay-related decisions in subjects with ADHD. For this reason, we manipulated the feedback trial length (7 feedback trials with a duration of 3, 15, or 45 seconds) and analyzed subsequent trial durations. Thus, the mean trial duration in seconds, which depended on previous feedback trial lengths, was used as an additional dependent variable.

#### Questionnaire of Current Motivation (QCM) [96]

The QCM is an 18-item questionnaire that was designed to assess current motivational aspects regarding task completion, including the dimensions of task interest, perceived challenge, mastery confidence, and incompetence fear, according to the cognitive-motivational process model [97]. Subjects are asked to evaluate the items on a seven-point Likert rating scale (from 1 =  disagree to 7 =  agree) and higher scores reflect higher task-related motivation. As differences in education history (subjects with ADHD more often attend special schools, repeat grades, more often receive special educational services and are less likely to graduate from school [98], [99]), self-esteem, and control beliefs (subjects with ADHD have a more external locus of control and suffer from lower self-esteem [100]–[102]) may differentially affect task-related motivation in both experimental groups, the QCM was considered to be a possible covariate of interest in the present study in order to rule out systematic motivational group differences which might already have existed prior to the experimental manipulation of reward options in the present study and which might independently impact the dependent variables. The prognostic value of the QCM was demonstrated by showing that mastery confidence and incompetence fear predicted learning performance in a complex, computer-simulated system [97]. Moreover, the task properties (self-regulated vs. question guided) and characteristics of the subject (“slow learners” vs. “fast learners”) were demonstrated to mediate the association between task interest and challenge with learning success [96]. In this study, we adopted the following QCM items: (1) “I enjoy doing this kind of task.” (2) “The task seems very interesting to me.” (3) “I'm very curious about how well I will perform the task.” (4) “I'm a little bit scared that I could make a fool of myself.” For each task, the subjects answered these four items subsequent to the practice trial, and the individual mean score, with item four being inverted, served as an indicator of the potential motivational group differences in task engagement.

### Procedure

The study was conducted at the Department of Psychiatry and Psychotherapy, University of Rostock. After explaining the general study purpose (the study was introduced as an examination of differences in attention, memory, and time perception between subjects with and without ADHD), the study procedure was explained, and the subjects underwent IQ testing. Subsequently, the subjects were seated in a quiet and dimmed experimental room and worked on the experimental tasks in the following order: delay aversion task, time reproduction task, n-back task, stop-signal task, time discrimination task, and CPT. For all tasks, a global instruction asked the subjects to perform the computerized tasks as fast and as accurately as possible. Each subsequent task was introduced by practice trials.

Additionally, subjects who participated in the reward condition were given the opportunity to earn extra money, depending on their performance quality. In doing so, reward conditions for each single task were explained after the subjects had performed the practice trials and after they had indicated their task-specific motivational ratings. The design of the reward options was guided by the aim of distributing overall gains homogeneously across tasks. In this way, we intended to prevent differences in the degree of reward-induced motivation between tasks. For three tasks, a response-cost option was used (stop-signal task: seed money of 0.50 €, +0.01 € for each correct answer and −0.01 € for each incorrect answer, +0.03 € for each correct inhibition and −0.03 € for each incorrect inhibition, CPT: seed money of 0.50 €, +0.03 € for each correct answer and −0.03 € for each incorrect answer; n-back task: +0.01 € for each correct answer and −0.05 € for each incorrect answer). The reward conditions for the remaining tasks were as follows: time discrimination task, +0.10 € for each correct answer; delay aversion task, +0.10 € for trial duration ≥60 s, +0.08 € for ≥50 s, +0.06 € for ≥40 s, and +0.04 € for ≥30 s; time reproduction task, +0.10 € if the deviation between the demanded and the reproduced time interval was ≤20%. Assuming perfect performance, a maximum of 13.97 € could be gained across all tasks. Subjects in the reward condition were provided feedback on their earnings at the end of the task (stop-signal task, CPT, or time discrimination task) or they were provided trial-based feedback in addition to feedback at the end of the task (n-back task, time reproduction task, and delay aversion task). The reward feedback was contingent on the subject's task performance.

The tasks were projected onto a white screen using a Sanyo LCD Z2 projector. The subject's distance to the screen was approximately 138 inches, and the screen was 51 inches wide. The visual angle was 21 degrees. The experimental paradigm was presented using Presentation software (Neurobehavioral Systems), which was installed on a desktop PC (Compaq HP dc 5700; CPU: Pentium 4.3 GHz; 1 GB RAM). The total duration of the tests was approximately 2.5 hours.

The study was performed in accordance with the latest version of the Declaration of Helsinki. The ethics committee of the Faculty of Medicine of the University of Rostock specifically approved this study, and written informed consent was received from all participants. At the beginning of the study, all of the subjects were told that they would be compensated for their time and expense with 20 €. Additionally, the subjects in the reward condition were told that they could earn extra money depending on the quality of their performance. To show equal appreciation for the effort of all subjects, however, all subjects received 30 € after finishing the study, irrespective of their performance quality. This procedure was already applied by other authors [103] (C. Bidwell, personal communication, September 28, 2012).

### Statistical analyses

Data were analyzed using SPSS version 17 (SPSS Inc., Chicago, IL, USA). Group comparisons for sociodemographic and clinical data were performed using univariate analyses of variance (ANOVAs). Because of significant group differences in age, age was included as a covariate in all statistical analyses. Group comparisons for task-related motivations were also performed using univariate ANCOVAs. Group differences in the dependent variables were analyzed using univariate analyses of covariance (time discrimination task), multivariate analyses of covariance (stop-signal task, CPT), and repeated-measures ANCOVAs with the diagnostic group and motivational condition as between-subject factors and the n-back level (n-back task), the seven time intervals (time reproduction task), and the first and second half of the test (delay aversion task) as within-subject factors. For repeated measurement analyses, tests for significance were performed using Pillai's trace statistic, and in case of significant main or interaction effects, post-hoc comparisons were performed by two-tailed univariate ANCOVAs, as well as independent-sample and paired-sample t-tests. The significance level for all tests was p≤0.05. The partial eta-squared (*η*_p_^2^) is reported as a measurement of effect size. Prior to analysis, raw data were z-transformed to identify extreme outliers (z>3.0), which were replaced by the respective group mean. The data are available at hpps://www.researchgate.net/profile/Ivo_Marx/.

## Results

### Task-Related Motivation

The initial task motivation for the six different tasks ranged from 3.71 to 5.13, which reflects an intermediate motivational baseline across the four experimental groups. Univariate ANCOVAs revealed a significant effect for the diagnostic group (*F*(1,73) = 7.10, *p* = .009) and the reward condition (*F*(1,73) = 7.05, *p* = .01) for the n-back task. The controls exhibited higher QCM-scores than the ADHD subjects, and the subjects in the non-reward condition exhibited higher QCM-scores than the subjects in the reward condition. Therefore, individual QCM-scores were included as covariates in n-back analyses. In all other tasks, no significant effects emerged (*p*>.05). QCM-scores for the initial task motivations are presented in Table 3.

**Table 3:** QCM Scores for Initial Task Motivation.

	ADHD R−	ADHD R+	CON R−	CON R+	Diagnostic Group	Reward	Diagnostic Group X Reward
	**(n = 18)**	**(n = 20)**	**(n = 20)**	**(n = 20)**			
	M (SD)	M (SD)	M (SD)	M (SD)		F	
**SST**	4.38 (1.00)	4.47 (1.32)	5.03 (1.09)	4.66 (1.10)	2.63	0.26	0.80
**n-back**	4.22 (1.06)	3.71 (1.19)	5.13 (1.47)	4.23 (0.88)	7.10**	7.05*	0.55
**CPT**	4.40 (1.26)	4.61 (1.35)	4.99 (1.16)	4.49 (0.89)	0.74	0.30	1.77
**Time Discrimination**	4.26 (1.09)	4.38 (1.21)	5.03 (1.11)	4.23 (0.98)	1.50	1.91	3.34
**Time Reproduction**	4.08 (1.01)	4.41 (1.25)	4.78 (1.28)	4.18 (0.93)	0.79	0.28	3.28
**Delay Aversion**	4.22 (1.27)	4.63 (1.31)	4.47 (1.10)	3.98 (0.93)	0.57	0.03	2.91

ADHD, ADHD group; CON, control group. R−, no financial reward; R+, financial reward. Diagnostic Group, main effect of diagnostic group; Reward, main effect of reward condition; Diagnostic Group × Reward, interaction effect of diagnostic group and reward condition. n, number of subjects; M, median; SD, standard deviation. SST, stop-signal task; n-back, n-back task; CPT, continuous performance task. F = F-Values.*p<0.05; **p<0.01; ***p<0.001.

### Task Performance

#### Stop-Signal Task

MANCOVA revealed a global effect for the diagnostic group (*F*(5,64) = 2.34, *p* = 0.05), and univariate post-hoc ANCOVAS revealed a significant between-subjects effect for the number of omission errors (*F*(1,68) = 7.39, *p* = .008, *η*_p_^2^ = 0.10), which was elevated in the ADHD subjects (CON: *M* = 5.51, *SD* = 7.04; ADHD: *M* = 12.42, *SD* = 14.81). No further significant effects emerged (*p*>.05). A post-hoc analysis was conducted to explore whether SSRT was abnormally prolonged relative to RT in the ADHD group, but no significant effects emerged (*p*>.05). Incentives did not affect performance in the stop-signal task (*p*>.05).

#### N-back Task

A significant between-subjects effect for d′ emerged (*F*(1,71) = 20.04, *p*<.001, *η*_p_^2^ = 0.22), with ADHD subjects impaired in both the 1-back (*F*(1,71) = 12.35, *p* = .001, *η*_p_^2^ = 0.15) and the 2-back condition (*F*(1,71) = 11.84, *p* = .001, *η*_p_^2^ = 0.14). Incentives did not affect performance in the n-back task (*p*>.05).

#### CPT

MANCOVA revealed a global effect for the diagnostic group (*F*(4,70) = 4.21, *p* = .004), and univariate post-hoc ANCOVAs exhibited elevated SDRT in the ADHD subjects (*F*(1,73) = 5.30, *p* = .02, *η*_p_^2^ = 0.07). Furthermore, we observed a global diagnostic group × reward condition interaction (*F*(4,70) = 3.02, *p* = .02), which was significant for FP (*F*(1,73) = 5.40, *p* = .02, *η*_p_^2^ = 0.07) and MDRT (*F*(1,73) = 4.38, *p* = .04, *η*_p_^2^ = 0.06). Post-hoc ANCOVAs revealed a higher prevalence of FP in the non-reward condition in ADHD subjects compared with the controls (*F*(1,35) = 6.89, *p* = .01, *η*_p_^2^ = 0.16), whereas no group differences were observed in the reward condition (*F*(1,37) = 0.06, *p* = .81, *ns*). With regard to MDRT, subjects with ADHD exhibited prolonged MDRT in the reward condition than in the non-reward condition (*t*(36) = 2.00, *p* = .05), but no such effect was observed in the controls (*t*(38) = 0.70, *p* = .49, *ns*).

#### Time Discrimination

No significant effects emerged for the sensitivity threshold (*p*>.05). To determine whether performance accuracy in the ADHD group was correlated with slower and more variable reaction times, which could be interpreted as the need for an additional effort to make correct decisions [57], we further analyzed our data with respect to RT and SDRT. We observed an effect of reward condition on RT (*F*(1,73) = 7.72, *p* = .007, *η*_p_^2^ = .10) and SDRT (*F*(1,73) = 11.57, *p* = .001, *η*_p_^2^ = .14); all subjects were faster and less variable in their decision times in the reward condition compared with the non-reward condition (RT: non-reward condition: *M* = 855.0 ms, *SD* = 294.39 ms; reward condition: *M* = 693.04 ms, *SD* = 249.70 ms; SDRT: non-reward condition: *M* = 845.60 ms, *SD* = 487.54 ms; reward condition: *M* = 536.89 ms, *SD* = 289.15 ms). Overall, no differences emerged between the experimental groups (*p*>.05); the incentives affected task performance for all subjects.

#### Time Reproduction

Absolute discrepancy scores were elevated in subjects with ADHD (*F*(1,72) = 14.66, *p*<.001, *η*_p_^2^ = 0.17). Absolute discrepancy scores increased as the time interval to be reproduced increased in length (*F*(6,432) = 3.57, *p* = .002, *η*_p_^2^ = .05). Furthermore, the absolute discrepancy scores increased more in the ADHD group (*F*(6,432) = 5.49, *p*<.001, *η*_p_^2^ = .07), and there was a significant interaction among the absolute discrepancy score increase, block order, and the reward condition (*F*(6,432) = 2.49, *p* = .02, *η*_p_^2^ = .03). However, post-hoc repeated measures ANCOVAs revealed no significant interactions between the increase in absolute discrepancy score and block order under both reward conditions (*p*>.05). Additionally, we observed a significant increase in the absolute discrepancy score with the diagnostic group and the reward condition (*F*(6,432) = 3.58, *p* = .002, *η*_p_^2^ = .05). Post-hoc repeated measures ANCOVAs revealed larger absolute discrepancy score increases in the ADHD group than in the control group in the non-reward condition (*F*(6,204) = 6.35, *p*<.001, *η*_p_^2^ = .16) but not in the reward condition (*F*(6,222) = 0.94, *p* = .47, *ns*). Consequently, the mean absolute discrepancy score across all time intervals differed significantly between the groups in the non-reward condition (*F*(1,34) = 19.00, *p*<.001, *η*_p_^2^ = .36) but not in the reward condition (*F*(1,37) = 1.80, *p* = .19, *ns*).

The accuracy coefficient scores were lower in the reward condition than in the non-reward condition (*F*(1,72) = 4.81, *p* = .03, *η*_p_^2^ = .06). Furthermore, there was a significant interaction between the diagnostic group and block order (*F*(1,72) = 4.91, *p* = .03, *η*_p_^2^ = .06), and post-hoc analyses revealed a decrease in accuracy coefficient scores in the second task block compared with the first task block in ADHD subjects (*t*(37) = 2.71, *p* = .01) but not in controls (*t*(38) = 0.22, *p* = .82, *ns*).

#### Delay Aversion

The overall mean trial duration differed significantly depending on the diagnostic group (*F*(1,72) = 10.73, *p* = .002, *η*_p_^2^ = .13) and reward condition (*F*(1,72) = 8.92, *p* = .004, *η*_p_^2^ = .11), with ADHD subjects and subjects in the non-reward condition exhibiting shorter time intervals. Univariate ANCOVA with post-hoc comparisons revealed that ADHD subjects in the reward condition did not demonstrate different performance compared with controls in the non-reward condition (*F*(3,72) = 6.76, *p*<.001, ADHD, reward condition vs. CON, non-reward condition: *p* = 1.00, *ns*). Repeated-measures ANCOVA revealed no effect of imposing post-trial delays of different lengths on subsequent interval lengths (*p*>.05).

**Figure 1:**
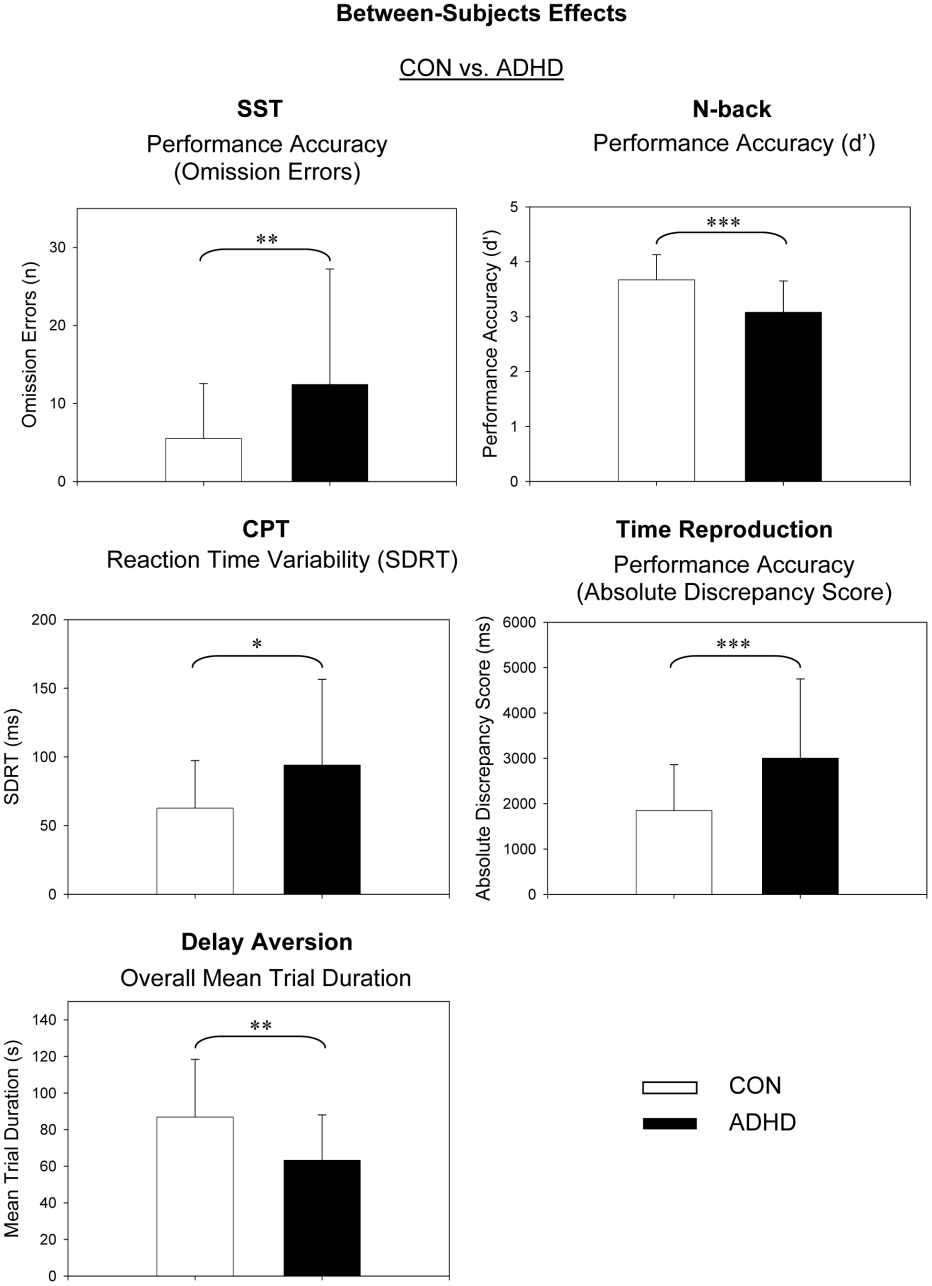
Between-Subjects Effects: Controls vs. ADHD. CON, control group; ADHD, ADHD group. SST, stop-signal task; CPT, continuous performance task. d′, discriminability score; SDRT, standard deviation of reaction time. n, number; ms, milliseconds; s, seconds. *p <.05, **p<.01, ***p<.001.

**Figure 2:**
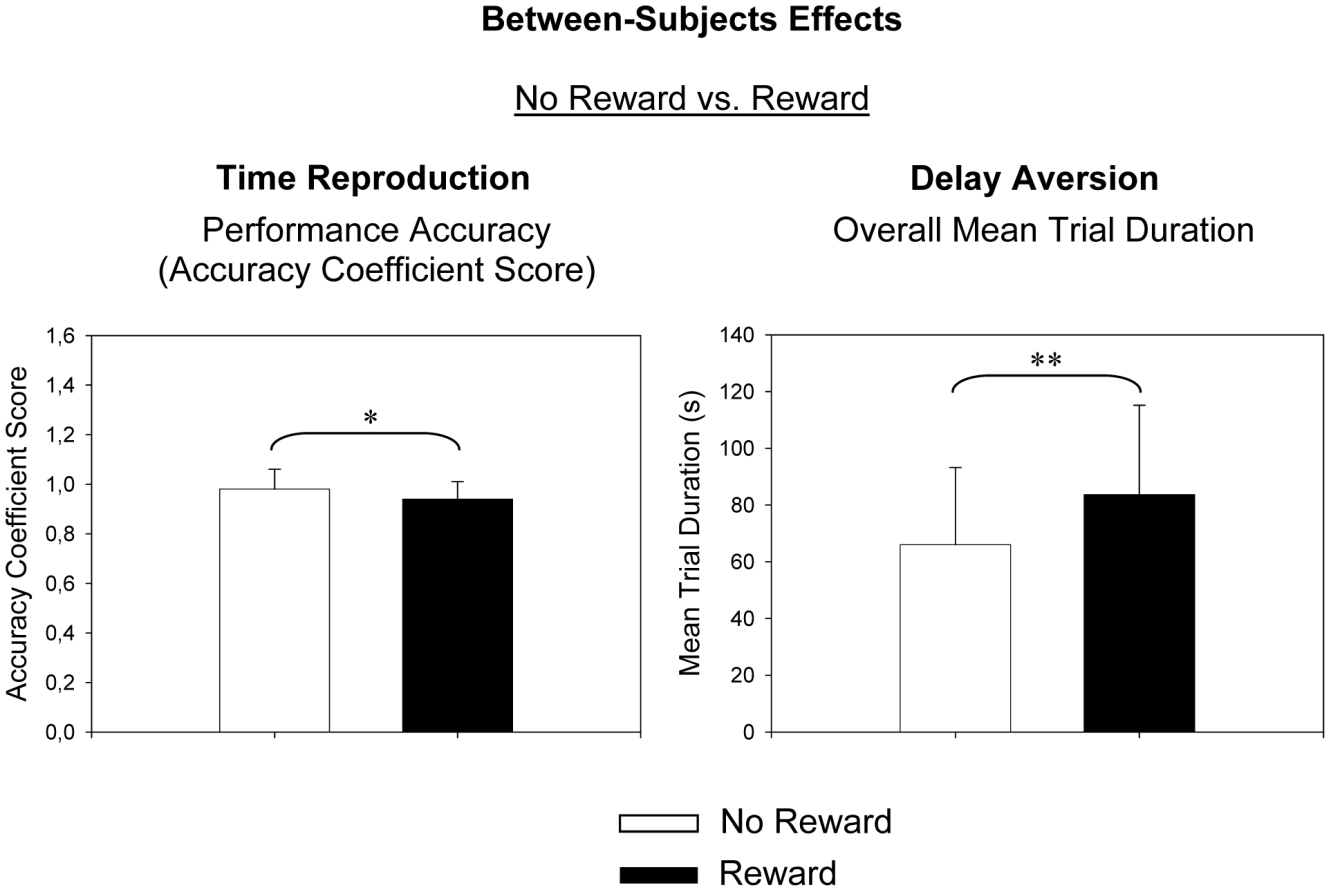
Between-Subjects Effects: No Reward vs. Reward. s, seconds. *p<.05, **p<.01, ***p<.001.

**Figure 3:**
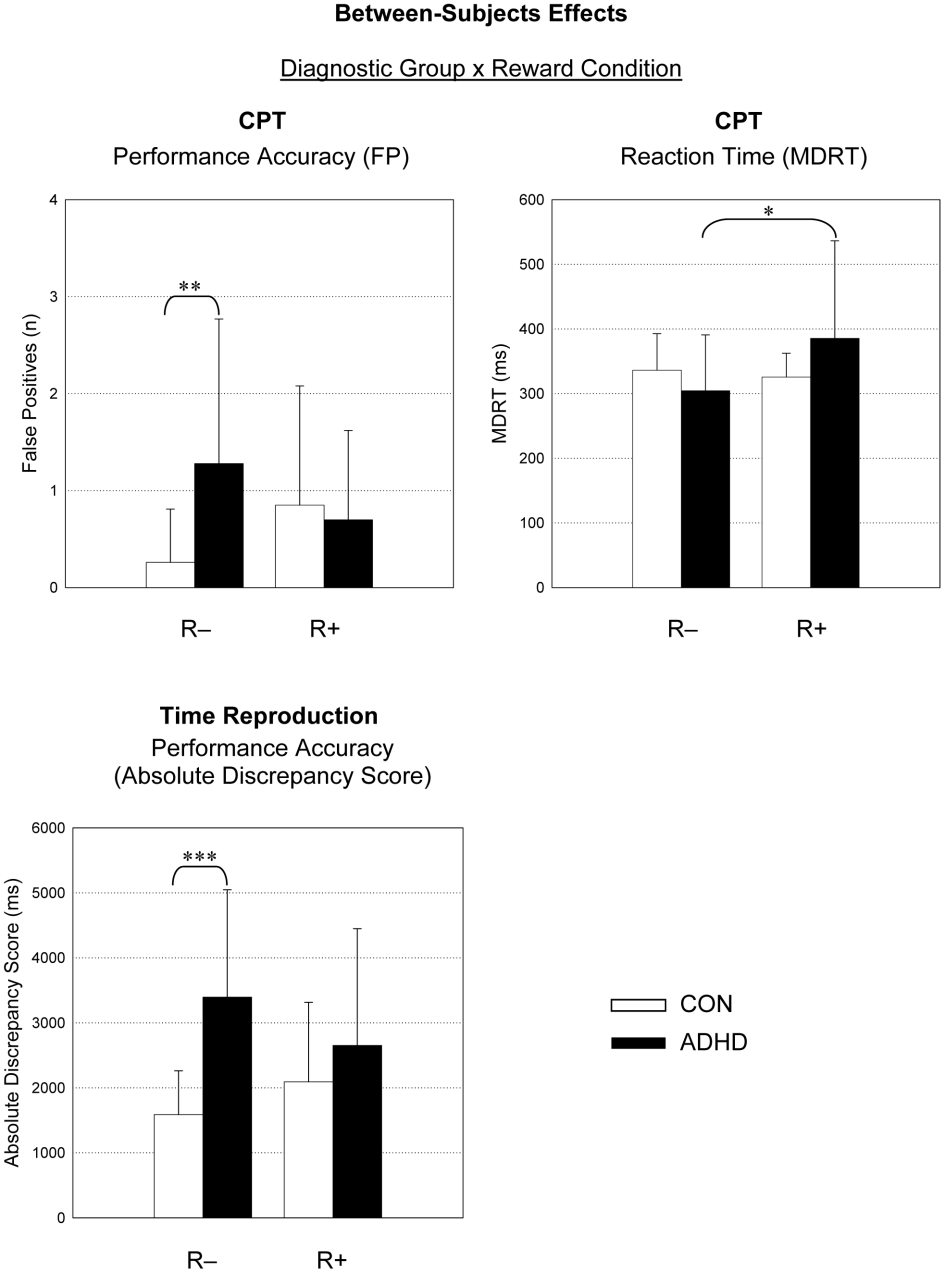
Between-Subjects Effects: Diagnostic Group x Reward Condition. CPT, continuous performance task. FP, false positives; MDRT, median reaction time. R−, non-reward condition; R+, reward condition; n, number; ms, milliseconds. *p<.05, **p<.01, ***p<.001.

**Figure 4:**
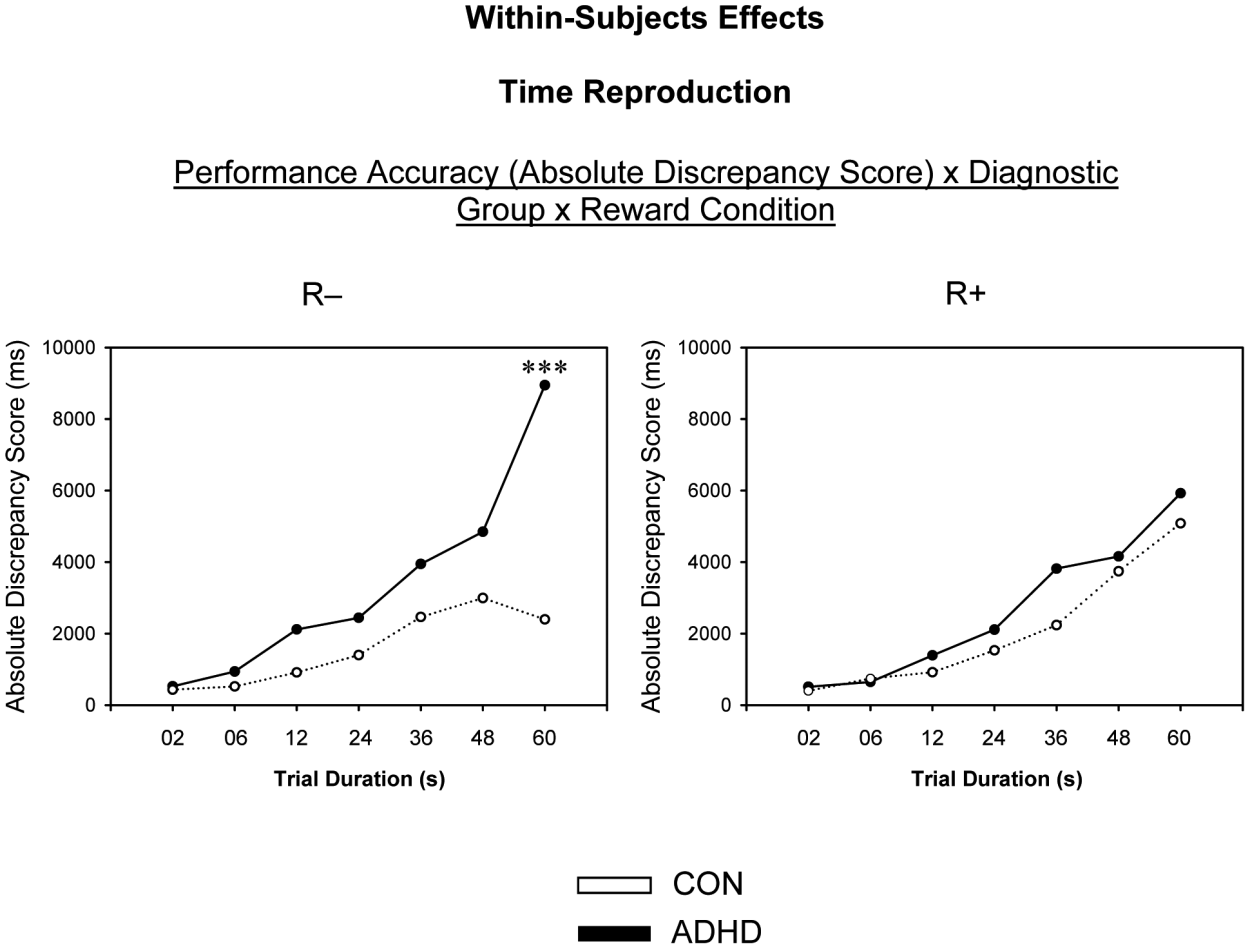
Within-Subjects Effects: Time Reproduction. R−, non-reward condition; R+, reward condition; ms, milliseconds. *p<.05, **p<.01, ***p<.001.

No further between- and within-subject main effects or interaction effects were observed (*p*>0.05). The means and standard deviations for all dependent measures can be derived from Table 4. Significant between-subject effects are presented in Figure 1 (diagnostic groups), Figure 2 (reward conditions) and Figure 3 (interaction effects), and significant within-subject effects are presented in Figure 4.

**Table 4:** Measures of Task Performance.

	ADHD R−	ADHD R+	CON R−	CON R+	Diagnostic Group	Reward	Diagnostic Group × Reward
	**(n = 18)**	**(n = 20)**	**(n = 20)**	**(n = 20)**			
	M (SD)	M (SD)	M (SD)	M (SD)		F	
**SST**							
** MDRT**	448.66 (97.39)	436.38 (103.89)	438.15 (78.59)	422.85 (82.32)	0.00	0.62	0.00
** SDRT**	126.28 (44.19)	113.44 (45.00)	118.09 (75.86)	95.06 (28.91)	0.62	2.35	0.15
** SSRT**	282.12 (107.81)	265.48 (115.33)	239.61 (66.83)	247.27 (57.42)	1.03	0.09	0.40
** Hits**	57.44 (28.04)	57.33 (31.83)	67.42 (38.23)	54.44 (35.39)	0.43	0.77	0.61
** OE**	13.56 (17.84)	11.28 (11.42)	5.58 (9.14)	5.44 (4.08)	7.39**	0.14	0.12
**n-back**							
** RT**	726.74 (215.06)	735.50 (195.92)	596.70 (170.42)	689.83 (162.75)	1.37	0.42	0.88
** d′**	2.97 (0.52)	3.19 (0.61)	3.75 (0.44)	3.60 (0.48)	20.04***	0.92	1.75
**CPT**							
** FP**	1.28 (1.49)	0.70 (0.92)	0.26 (0.55)	0.85 (1.23)	2.96	0.00	5.40*
** OE**	6.94 (15.66)	1.30 (1.49)	0.50 (1.00)	0.63 (0.67)	3.00	2.76	2.95
** MDRT**	304.45 (86.41)	385.56 (150.78)	336.17 (56.66)	325.66 (36.80)	0.00	2.26	4.38*
** SDRT**	90.48 (67.62)	97.24 (59.02)	69.24 (43.65)	56.26 (21.56)	5.30*	0.12	0.66
**Time Discrimination**							
** ST**	217.08 (61.88)	192.38 (57.33)	229.00 (213.02)	168.88 (62.78)	0.04	2.38	0.42
** MRT**	860.52 (260.44)	703.29 (258.78)	850.03 (328.68)	682.79 (246.56)	0.98	7.72**	0.00
** SDRT**	801.39 (424.80)	528.04 (249.06)	885.39 (545.81)	545.75 (330.78)	0.57	11.57***	0.10
**Time Reproduction**							
** ERR**	3394.93	2651.88	1587.36	2092.64	14.66***	0.08	3.56
	(1653.16)	(1796.32)	(675.55)	(1222.55)			
** AS**	0.96 (0.08)	0.94 (0.09)	1.00 (0.07)	0.95 (0.05)	2.77	4.81*	0.78
**Delay Aversion**							
** DUR**	55.46 (25.07)	70.24 (22.92)	76.00 (25.84)	97.14 (33.65)	10.73**	8.92**	0.20

ADHD, ADHD group; CON, control group. R−, no financial reward; R+, financial reward. Diagnostic Group, main effect of diagnostic group; Reward, main effect of reward condition; Diagnostic Group × Reward, interaction effect of diagnostic group and reward condition. n, number of subjects; M, median; SD, standard deviation. SST, stop-signal task, n-back, n-back task, CPT, continuous performance task; MDRT, median reaction time in the “go”-trials (SST); SDRT, reaction time variability in the “go”-trials (SST); SSRT, stop-signal reaction time (SST); Hits, number of correct inhibitions (SST); OE, omission errors (SST); RT, reaction time for correctly identified targets in milliseconds (n-back); d′, performance accuracy (n-back); FP, false positives (CPT); MDRT, median reaction time for correctly identified targets in milliseconds (CPT); SDRT, standard deviation of reaction time for correctly identified targets in milliseconds (CPT); ST, sensitivity threshold in milliseconds (Time Discrimination); ERR, absolute value of the deviation between specified and produced time interval in milliseconds (Time Reproduction); AS, Accuracy Score (Time Reproduction); DUR, mean trial duration in seconds (Delay Aversion). F, F-value.*p<0.05; **p<0.01; ***p<0.001.

### Cumulative Earnings

Across all tasks, controls gained slightly more money (*M* = 8.49±2.47 €; range: 4.49–12.54 €) than ADHD subjects (*M* = 7.13±2.35 €; range: 2.46–11.48 €), but this difference was not significant (*F*(1,31) = 2.04, *p* = .16, *ns*).

## Discussion

The aim of the present study was to examine the motivating effect of financial reward contingencies on task performance in adults with ADHD. We hypothesized that subjects with ADHD would exhibit performance deficits in all applied tasks in the absence of reward but that they would improve their performance in anticipation of a reward. The improvement was anticipated to be most pronounced in the domains of inhibition (stop-signal task) and time perception (time reproduction task).

As expected, impaired performance was observed across several cognitive domains in subjects with ADHD, including stop-signal task omission errors, n-back accuracy, reaction time variability in the CPT, and time reproduction accuracy. Moreover, subjects with ADHD demonstrated shorter time intervals in the delay aversion task. However, although our data are consistent with recent findings of increased delay aversion in adults with ADHD [28], the shorter trial durations in the current study cannot definitely be interpreted in terms of increased delay aversion because of ceiling effects in the implemented task. Subjects with ADHD exhibited optimal performance; they produced trial durations of about one minute, and waiting beyond this period of time was no longer rewarded. It is possible that the task was too easy to provoke marked delay aversion in subjects with ADHD.

With regard to the domains that were impaired in subjects with ADHD in the present study, reward affected the task performance in different ways. Whereas stop-signal omission errors, n-back accuracy and response time variability in the CPT were not affected by rewards, a differential group effect was observed for time reproduction. Subjects with ADHD exhibited higher absolute discrepancy scores with increasing time interval lengths and, as a consequence, a higher overall absolute discrepancy score only in the non-reward condition but not in the reward condition. Thus, reward minimized absolute discrepancy score increases in subjects with ADHD and equalized the performance between the groups. Likewise, McInerney and colleagues [58] found time reproduction performance accuracy to normalize in the reward condition in children with ADHD. With regard to the delay aversion task performance, all subjects exhibited decreased delay aversion in the presence of reward, and subjects with ADHD in the reward condition did not differ from controls in the non-reward condition.

Furthermore, reward affected task performance in cognitive domains where subjects with ADHD were not impaired. A differential group effect was observed for CPT performance; subjects with ADHD exhibited longer reaction times in the reward condition than in the non-reward condition, whereas controls did not. In addition, ADHD subjects exhibited a higher number of false positives in the non-reward condition but not in the reward condition. Thus, it may be concluded that subjects with ADHD, when rewarded, take more time to make their decisions, which results in a lower number of impulsivity errors as measured by false positives. Similarly, a speed-accuracy trade-off for the stop-signal task was reported by Scheres et al. [73] in children with ADHD. In the time reproduction task, reward negatively affected task performance because all subjects, irrespective of diagnosis, exhibited decreased time reproduction accuracy coefficient scores in the reward condition, which indicates that they under-reproduced the presented time intervals.

In accordance with recently conducted studies, we determined that subjects with ADHD were not impaired with respect to the number of successful inhibitions [47], [49]–[52], but that they committed more omission errors than the controls in the stop-signal task [47]. Although our performance accuracy results are consistent with the literature, we observed no group differences in speed measurements, especially larger reaction time variability and prolonged SSRT, which were previously indicated by meta-analyses [46]. The lack of a significant effect is not likely the result of the gender or ADHD subtypes in the ADHD group, which were similar to those of other studies. However, because the subjects with ADHD scored only 0.8 SD above the mean on the hyperactive-impulsive symptoms subscale in our study, the lack of hyperactive-impulsive symptom severity may have contributed to these results.

In line with the literature, we further found decreased performance accuracy in the CPT in the absence of reward [11], [18], [19], [21]; however, we failed to identify increased reaction times in the n-back task as indicated by Bayerl and colleagues [54]. In the current study, like in the study conducted by Marx et al. [28], subjects with ADHD were impaired in n-back performance accuracy but did not display prolonged reaction times. In contrast, two other studies found performance accuracy to be unimpaired but they failed to analyze reaction times [48], [53], and another study found reaction time to be prolonged but they failed to analyze performance accuracy [54]. Thus, it might be speculated that n-back performance underlies a speed-accuracy trade-off in adult ADHD, as it had been demonstrated for the stop-signal task previously [73]. Future studies should address this issue by collecting both accuracy and speed measurements.

Furthermore, although studies in children and adolescents [55], [56], [104] as well as adults with ADHD [28] consistently report deficits in discriminating stimuli that differ in their presentation duration by only several milliseconds, no discriminatory deficits were observed in adults with ADHD in the present study. In contrast, the numerical values for the sensitivity threshold in subjects with and without ADHD are very similar to those reported for controls in the study by Marx et al. [28]. Similarly, another study [57] failed to identify differences in the number of correct decisions in adults with ADHD, but the authors observed qualitative differences in task completion between children and adults with ADHD. Children produced fewer correct discriminations but did not differ in reaction time and reaction time variability compared with control children, whereas adults exhibited higher and more variable reaction times but did not differ from controls in the number of correct discriminations. The authors interpreted this diverging pattern as evidence of an additional effort needed for correct decision making in adults. This additional effect may point to the development of compensatory strategies throughout the course of a lifetime. Testing for group differences in reaction times and reaction time variability in our sample, however, did not identify any impairment in the ADHD group, which indicates that the subjects in our study did not exhibit any time discrimination deficit. Again, because performance quality was found to be associated with the extent of hyperactive-impulsive symptoms [55], the lack of group differences in the present study may be a result of a lack of hyperactive-impulsive symptom severity in the ADHD group.

Our results provide support for current models of ADHD that incorporate motivational pathways to the disorder. First, the cognitive-energetic model [8] suggests that motivation improves cognitive performance, as evidenced by our data. However, future studies should identify which specific cognitive functions are influenced by reward, how large these effects are, and what amount of reinforcement is necessary to achieve maximum performance in subjects with ADHD. Answering these questions will aid the understanding of the particular contribution of cognitive and motivational factors to the phenotype of the disorder and will help to suitably evaluate reinforcer quality and quantity to improve or even compensate for existing performance deficits.

With regard to the dual-pathway-model of ADHD [25], our data suggest a stronger-than-modeled linkage between the executive and the reward circuit that is mediated by strategy use contingent on reward. In this sense, highly valued reward contingencies may motivate subjects with ADHD to self-impose cognitive strategies in delay-rich settings, as suggested by the CPT performance in our study, such that delay-rich situations do not necessarily lead to poor task performance. Self-imposing cognitive strategies in delay-rich settings may help subjects with ADHD to learn to successively bridge even larger delays, thereby leading to repetitive mastery of delay-associated demands and, as a consequence, reducing delay aversion.

There are some limitations of our study. First, we examined subjects who visited the ADHD outpatient service of our outpatient clinic. As indicated by the symptom severity scores, the ADHD symptom severity was rather moderate. The relatively low symptom severity and the low rate of comorbid disorders could indicate that we included rather moderately affected subjects in our study. In addition, one of the tasks used (the delay aversion task) appears to display a ceiling effect. Therefore, the presence of group differences between subjects with and without ADHD in some of the domains, namely, response inhibition and time discrimination, should be replicated in samples with higher symptom severity. In addition, a more challenging task for delay aversion may be needed so that the resultant findings can be interpreted in terms of marked delay aversion. Second, it is possible that the 20 € compensation for study participation increased the initial degree of motivation such that the additional reward produced a performance increase in addition to this effect. For this reason, it is possible that the motivational power of financial reward was not as strong as it could have been with non-compensated study participation or if larger rewards were used. Third, as the order of experiments in our study was not randomized, order effects cannot be ruled out. Fourth, we implemented reward and response cost options, which makes it difficult to directly compare the performance-increasing effect of both contingencies. Because there is some evidence that both contingencies may be differentially effective [105], further studies should either maintain or systematically manipulate the type of reward contingency. Another point that has to be discussed is the reward valence. As it is intuitive that personal relevance is essential for reward efficacy, future studies should systematically examine which motivational factors others than financial reward may be (more) effective in improving performance, particularly with respect to individuals of different ages. This issue has implications both for research, as effective reinforcers are needed to examine reward-induced increases in cognitive performance, and for therapeutic settings, as the success of behavior modification efforts depends on reinforcer adequacy. In this context, monetary rewards have already been demonstrated to affect performance more strongly than social rewards in subjects with ADHD [106]. Finally, as a substantial number of statistical group comparisons were computed in the current study, the possibility cannot be ruled out that some of the findings may have been identified due to chance.

## Conclusions

By implementing a large test battery that covered different key domains of cognitive functioning associated with ADHD, the current study succeeded in reproducing cognitive dysfunctions typical of children and adults with ADHD and demonstrated for the first time that the cognitive functioning of adults with ADHD is susceptible to reward. Forging a bridge to current models of ADHD, our results suggest the existence of primarily motivational deficits in delay aversion and time reproduction, with rewards equalizing the performance of subjects with and without ADHD. Furthermore, our data suggest cognitive strategies of “stopping and thinking” as possible underlying mechanisms for performance improvements that seem to be mediated by reward (CPT), thereby highlighting the interaction between motivation and cognition in ADHD.

Future studies are needed to identify domains that may be influenced by reward (identification of cognitive vs. motivational deficits), the extent to which influence is possible (effect size of reward), and finally, the quality and amount of reinforcement necessary to achieve maximum performance in subjects with ADHD (improvement vs. compensation). In addition to theoretical model specifications, this knowledge may help to determine which therapeutic interventions are most effective in different settings associated with different cognitive demands (mediation of cognitive strategies vs. motivational incentives).
